# Supplementary material for: A Double-Deck Structure of Reduced Graphene Oxide Modified Porous Ti3C2Tx Electrode towards Ultrasensitive and Simultaneous Detection of Dopamine and Uric Acid
Source: Biosensors (Basel). 2021 Nov 18;11(11):462. doi: 10.3390/bios11110462 (PMC8615994; doi:10.3390/bios11110462)
Supplement: Supplementary file 1 [file biosensors-11-00462-s001.zip › biosensors-1437418-SI.pdf]

Supplementary Materials

A Double-Deck Structure of Reduced Graphene Oxide Modified Porous $Ti_3C_2T_x$ Electrode towards Ultrasensitive and Simultaneous Detection of Dopamine and Uric Acid

Yangguang Zhu [1,2], Qichen Tian [3], Xiufen Li [1,*], Lidong Wu [4], Aimin Yu [5], Guosong Lai [6], Li Fu [7], Qiuping Wei [8], Dan Dai [2,9], Nan Jiang [2,9], He Li [2,9], Chen Ye [2,9,*] and Cheng-Te Lin [2,9,*]

1 Laboratory of Environmental Biotechnology, School of Environmental and Civil Engineering, Jiangnan University, Wuxi 214122, China; zhuyangguang@nimte.ac.cn
2 Key Laboratory of Marine Materials and Related Technologies, Zhejiang Key Laboratory of Marine Materials and Protective Technologies, Ningbo Institute of Materials Technology and Engineering (NIMTE), Chinese Academy of Sciences, Ningbo 315201, China; daidan@nimte.ac.cn (D.D.); jiangnan@nimte.ac.cn (N.J.); lihe@nimte.ac.cn (H.L.)
3 College of Chemical Engineering, Zhejiang University of Technology, Hangzhou 310032, China; tianqichen@nimte.ac.cn
4 Key Laboratory of Control of Quality and Safety for Aquatic Products, Chinese Academy of Fishery Sciences, Beijing, 100141, China; wulidong19849510@hotmail.com
5 Department of Chemistry and Biotechnology, Faculty of Science, Engineering and Technology, Swinburne University of Technology, Hawthorn, VIC 3122, Australia; aiminyu@swin.edu.au
6 Department of Chemistry, Hubei Normal University, Huangshi 435002, China; gslai@hbnu.edu.cn
7 College of Materials and Environmental Engineering, Hangzhou Dianzi University, Hangzhou 310018, China; fuli@hdu.edu.cn
8 School of Materials Science and Engineering, Central South University, Changsha 410083, China; qiupwei@csu.edu.cn
9 Center of Materials Science and Optoelectronics Engineering, University of Chinese Academy of Sciences, Beijing 100049, China
* Correspondence: xfli@jiangnan.edu.cn (X.L.); yechen@nimte.ac.cn (C.Y.); linzhengde@nimte.ac.cn (C.-T.L.)

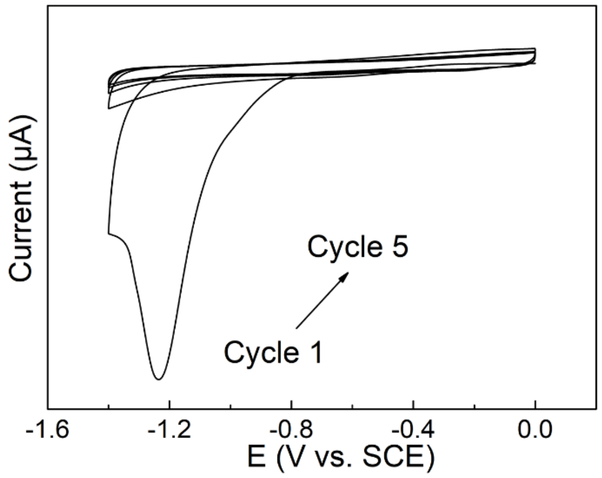

Figure S1. CV curves of $Ti_3C_2T_x$/rGO electrode on electrochemical reduction of GO to rGO.

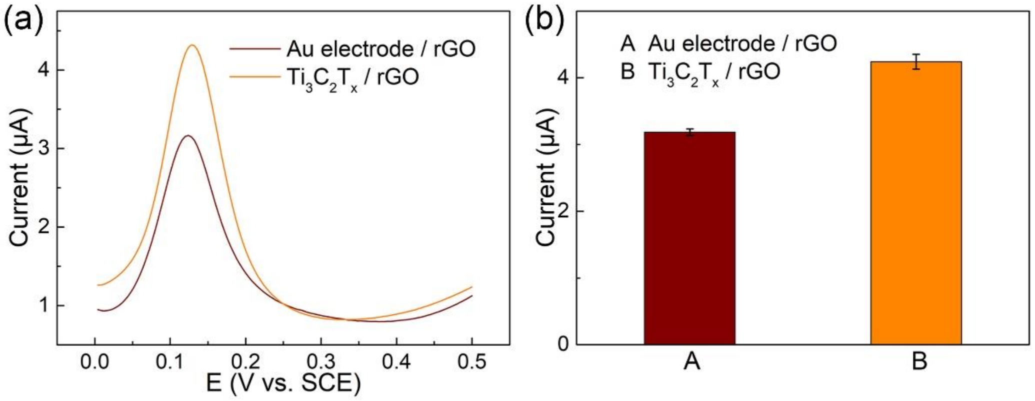

Figure S2. (a) Performance comparison of DPV curves on $Ti_3C_2T_x$/rGO and Au/rGO electrode with 10 μM DA in PBS. (b) The corresponding current value of (a).

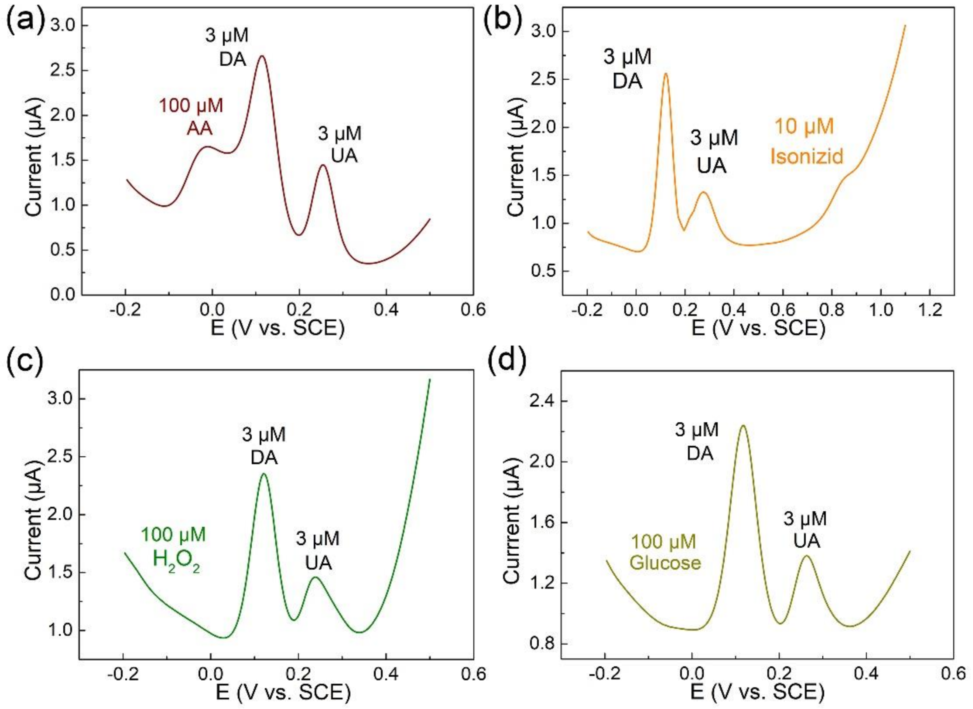

Figure S3. The anti-interference of our electrode in the presence of 100 μM glucose, 100 μM ascorbic acid, 100 μM H_2O_2 and 10 μM isoniazid with PBS containing 3 μM DA and 3 μM UA.

Table S1. The fitting parameters of EIS for GCE, $Ti_3C_2T_x$ and $Ti_3C_2T_x$/rGO electrode.

Electrode	R_s (Ω)	Q_{coat} (F)	R_p (Ω)	Q_{sub} (F)	R_{ct} (Ω)
GCE	181.1	1.49×10^{-6}	2052	2.84×10^{-3}	1036.0
$Ti_3C_2T_x$	210.0	1.68×10^{-6}	2209	2.52×10^{-3}	628.8
$Ti_3C_2T_x$/rGO	221.4	1.50×10^{-4}	596.4	3.83×10^{-3}	369.6
